# Diagnostic performance of the microRNA‐371a assay in small testicular masses: a real‐world cohort study

**DOI:** 10.1111/bju.70215

**Published:** 2026-03-13

**Authors:** Felix Seelemeyer, David Pfister, Ruben Gößmann, Olivia Steenbock, Roberto Pappesch, Sabine Merkelbach‐Bruse, Axel Heidenreich

**Affiliations:** ^1^ Uniklinik Cologne, Department of Urology Cologne Germany; ^2^ Uniklinik Cologne, Department of Pathology Cologne Germany

**Keywords:** small testicular mass, microRNA‐371a‐3p, testicular cancer, orchidectomy, ablatio testis, biomarker, seminoma, non‐seminoma

## Abstract

**Objectives:**

To evaluate the diagnostic performance of the Conformité Européenne [CE]‐certified microRNA‐371a‐3p (miR371a) assay in patients undergoing inguinal exploration for small testicular masses of unknown histology in a real‐world setting.

**Patients and Methods:**

Between March 2023 and March 2024, 60 patients underwent inguinal exploration for testicular masses of unknown histology. For miR371a analysis, peripheral venous blood was obtained and sent to the Department of Molecular Pathology within 30 min. The extracted miRNA was reverse‐transcribed into complementary DNA (cDNA) with miRNA‐specific stem‐loop primers, followed by a pre‐amplification step. Using quantitative polymerase chain reaction, the miR371a is quantified. The median crossing points of the triplicates was calculated and converted into a relative quantification value.

**Results:**

The evaluation of the miR371a assay showed a true‐positive result in 25 patients with seminoma. Eight patients with seminoma had false‐negative results. In the non‐seminoma group, six of seven (86%) patients had a true positive result. The false‐negative finding derived from a non‐seminomatous germ cell tumour (NSGCT) with predominant teratoma. The test was true‐negative in 16 patients with sarcoma, diffuse large B‐cell lymphoma, Leydig cell tumour, and Sertoli cell tumour. False‐positive results were obtained in four patients. The results yield a sensitivity of 77.5%, a specificity of 80% and a positive predictive value of 88.6% and negative predictive value of 64% for the miR371a test in our cohort. In the seminoma cohort, the sensitivity of the serum tumour markers was 12%. In the NSGCT cohort, the sensitivity was 66%.

**Discussion:**

In the absence of suitable markers, the miR371a assay shows promising results in the preoperative diagnosis of suspected localised small testicular tumour. Standardisation of pre‐analytical handling and prospective multicentre validation are essential to define its optimal role in preoperative decision‐making.

**Abbreviations**
AFP: α‐fetoprotein
AUC: area under the curve
CE: Conformité Européenne (European Conformity certification)
(T)GCT: (testicular) germ cell tumour
IQR: interquartile range
LDH: lactate dehydrogenase
mRNA: messenger RNA
miRNA/miR: micro‐RNA
NPV: negative predictive value
NSGCT: non‐seminomatous GCT
OR: odds ratio
PPV: positive predictive value
qPCR: quantitative PCR
ROC: receiver operating characteristic
RQ: relative quantification
STM: serum tumour marker

## Introduction

Testicular germ cell tumour (TGCT) is the most common solid malignant tumour among men aged 20–40 years. In most cases the suspicion of a GCT can be established by clinical examination, testicular ultrasonography, and the presence of elevated serum concentrations of tumour markers [1].

If GCTs are suspected, serum tumour markers (STMs) are determined before dissection of the affected testicle.

Only 50% of all TGCTs express one of the three commonly used serum markers: α‐fetoprotein (AFP), β‐hCG, and lactate dehydrogenase (LDH). Notably, seminomas lack AFP expression entirely [2].

However, one major drawback of the markers is their low overall sensitivity.

In any case of suspicious testicular masses with negative tumour markers, inguinal exploration with tumour enucleation and frozen‐section analysis should be performed to differentiate between benign and malignant masses [3]. In addition, it has been observed that the incidence of benign lesions increases by decreasing tumour size [4, 5, 6].

With the refinements of scrotal ultrasonography technique, incidentally detected small testicular neoplasms represent a clinical scenario of increasing frequency especially in men with fertility disorders [7]. Any diagnostic method with a high reliability to identify benign lesions will reduce the need for inguinal exploration and unnecessary orchidectomies.

MicroRNAs (miRNAs) are small, noncoding RNAs that post‐transcriptionally regulate gene expression by binding to the 30‐untranslated region of target messenger RNAs (mRNAs), resulting in mRNA degradation or translation repression [8].

In TGCT, miR371a‐3p represents a potential biomarker with diagnostic and predictive properties [9]. Several studies found that miR371a‐3p is upregulated in germ cell neoplasia *in situ*, seminoma, and non‐seminoma GCT (NSGCT) apart from teratoma [10].

It has been shown that miR371a‐3p predicts the presence of TGCT in primary testicular lesions of unknown histology with specificity of 89%, independent of the final histopathology for the orchidectomy specimen [11]. MiR371a‐3p also correlates with clinical stage and response to treatment [9].

The aim of this real‐world pilot study was to evaluate the diagnostic performance of the Conformité Européenne [CE]‐certified miR371a‐3p (miR371a) assay in a preoperative cohort of clinical Stage I testicular tumours, with a particular focus on small lesions as they typically present in routine clinical decision‐making.

## Patients and Methods

### Cohort and Study Design

In the 12‐month period from March 2023 to March 2024, 60 patients with suspicious testicular masses and clinical Stage I disease, without evidence of metastatic spread on CT or MRI, underwent inguinal exploration with either tumour enucleation or orchidectomy. Standard STMs (AFP <5.8 kU/L, β‐hCG <5 U/L, LDH <250 U/L) were determined preoperatively.

Patient characteristics and perioperative findings are summarised in Table 1.

**Table 1:** Patient characteristics (*n* = 60) and perioperative variables.

Variable	Value
Age, years, median (IQR)	36.5 (31.7–46.2)
Left side, *n*	25
Right side, *n*	35
pT1, *n*	33
pT2, *n*	7
Seminoma, *n*	33
Non‐seminoma, *n*	7
Other testicular masses, *n*	20
Diffuse large B‐cell lymphoma	1
Sarcoma	1
Inflammation	4
Haematoma	1
Fibrosis and atrophy	4
Sertoli cell tumour	2
Leydig cell tumour	4
Spermatocytic tumour	1
Adenomatoid tumour	1
Benign cystic mass	1
STM elevated, *n*	8
AFP only	1
β‐hCG only	3
AFP and β‐hCG	4
AFP, kU/L, median (IQR)	8.4 (2.5–24.6)
β‐hCG, U/L, median (IQR)	85.5 (12–216)
LDH, U/L, median (IQR)	203 (157–258)
STM within a normal range of in TGCT, *n*	32
Operation time, min, median (range)	44 (20–100)
Tumour size, cm, median (IQR)	2.4 (1.4–3.5)
Clavien–Dindo ≥III complications, %	0

A blood sample for miR371a assessment was collected within 12–24 h prior to surgery and again 48–72 h postoperatively to evaluate biomarker decline.

This single‐centre study was conducted at the Department of Urology, University Hospital Cologne. Preoperative miR371a assessments were part of a prospectively collected biobank protocol; the present analysis was performed retrospectively. The study was approved by the Ethics Committee of the University of Cologne, and written informed consent was obtained from all participants.

### Laboratory Procedures

The CE‐certified miR371a *in vitro* diagnostic assay (mir|detect GmbH, Bremerhaven, Germany) was used, and all blood samples were processed according to the manufacturer's protocol in the Department of Molecular Pathology at the University Hospital Cologne. Serum samples were collected in serum gel tubes (S‐Monovette®; Sarstedt AG & Co. KG, Nümbrecht, Germany) and subjected to a single‐spin centrifugation at 2500 ***g*** for 10 min at room temperature, typically within 30 min after blood draw; an internally defined upper limit of ≤2 h was maintained in accordance with published miRNA recommendations [12].

The miRNA extraction was performed on 200 μL of serum using the Maxwell RSC miRNA Plasma and Serum Kit (Promega Corp., Madison, WI, USA) without carrier RNA, with single lot numbers used per batch. Reverse transcription and pre‐amplification were carried out according to the CE‐certified mir|detect workflow, and real‐time quantitative PCR (qPCR) was performed on a Roche LightCycler 480 II system (Roche Diagnostics GmbH, Mannheim, Germany). Three technical replicates were run for both miR371a‐3p and the endogenous reference miR‐30b, which served as the internal normaliser.

The validated workflow included RNA isolation, complementary DNA synthesis, pre‐amplification, and qPCR‐based quantification. The LightCycler software calculated the median crossing point value (threshold cycle in PCR) from triplicates and converted it into a relative quantification (RQ) value. According to previously established analytical thresholds, RQ values ≥10 were considered positive, 5.1–9.9 indeterminate, and <5 negative. Although repeat testing is recommended for values within the 5–10 range, this was rarely feasible in our clinical workflow; therefore, all RQ values <10 were pragmatically classified as negative. Determination and validation of the analytical cut‐off values have been described elsewhere [9].

### Statistical Analysis

Continuous variables are reported as medians with interquartile ranges (IQRs) or ranges. Proportions are presented with 95% CIs calculated using the Wilson score method.

Associations between miR371a expression and tumour size or stage were examined using Kendall's Tau‐b correlation. Paired pre‐ and postoperative comparisons were evaluated using the Wilcoxon signed‐rank test. The Hodges–Lehmann estimator provided median paired differences with 95% CI.

Binary logistic regression models were constructed to evaluate miR371a status and tumour size as predictors of TGCT presence. Model performance was assessed using Nagelkerke's *R* ^2^; results are reported as odds ratios (ORs) with 95% CI. A two‐sided *P* < 0.05 was considered statistically significant.

Diagnostic accuracy of miR371a RQ values was assessed using receiver operating characteristic (ROC) analysis. The area under the curve (AUC) with 95% CI was calculated, and optimal cut‐off values were defined via the Youden index.

All statistical analyses were performed using the Statistical Package for the Social Sciences (SPSS), version 30.0 (IBM Corp., Armonk, NY, USA).

## Results

Histology revealed a seminomatous GCT or NSGCT in 40/60 (66%). The median (IQR) tumour size was 2.4 (1.4–3.5) cm with a range from 0.6 to 8.2 cm in patients with TCGTs.

MiR371a was truly positive in 25 patients in the seminoma group, while eight patients were diagnosed with a false‐negative result. The median (IQR) tumour size of patients with a false‐negative test was 1.4 (1.1–2.6) cm. In comparison, the median (IQR) tumour size in patients with miR371a‐positive seminoma was 2.9 (1.8–4.2) cm.

In the non‐seminoma group, five of six patients had a true‐positive result. Additionally, a patient with pure teratoma had a true‐positive result with an RQ value of 11.3.

There was a false‐negative result for non‐seminoma in a patient with a histology consisting of teratoma 85%, embryonal cell carcinoma 14%, yolk sac tumour 1%.

In the group of patients with non‐malignant lesions, four of 20 (20%) patients were tested positive: one patient with testicular purulent‐abscessing, destructive inflammation, one patient with testicular atrophy, and two patients with a Leydig cell tumour.

The sensitivity of the miR371a assay was 77.0% (95% CI 62.5–87.7%), the specificity was 80.0% (95% CI 58.4–91.9%), the positive predictive value (PPV) was 88.6% (95% CI 74.0–95.5%), and the negative predictive value (NPV) was 64% (95% CI 44.5–79.5%) for the miR371a test in our cohort (Table 2).

**Table 2:** Performance metrics for the miR371a assay.

miR371a assay	% (95% CI)
Sensitivity	77.5 (62.5–87.7)
Specificity	80.0 (58.4–91.9)
PPV	88.6 (74.0–95.5)
NPV	64.0 (44.5–79.5)

At diagnosis, eight patients with GCTs had positive STMs, all of whom also tested positive for miR371a. Among these, four had seminomas and four had NSGCTs. The sensitivity of STMs was 12% in the seminoma cohort and 66% in the NSGCT cohort.

There was a positive correlation between miR371a expression and tumour stage; however, the association was not statistically significant (*r* = 0.33, *P* = 0.054).

In addition, we analysed the correlation between the size of the testicular tumours and the level of miR371a. The analysis revealed a significant positive correlation between the miR371a RQ value and tumour size (Kendall's tau‐b: *r* = 0.417; *P* < 0.001; *n* = 39). We also analysed the predictive value of the miR371a test in the analysis of small testicular tumours, defined as a tumour size of <2 cm. Here, we still showed a sensitivity of 66%, a specificity of 80%, a PPV of 76%, and a NPV of 70%.

The ROC analysis demonstrated a high diagnostic performance of miR371a RQ value for predicting the presence of TGCT, with an AUC of 0.883 (95% CI 0.791–0.974; *P* < 0.001). The optimal cut‐off value determined by the Youden index was an RQ of 11.08, corresponding to a sensitivity of 77.5% and a specificity of 85%. In the subgroup of patients with tumours ≤2 cm, miR371a RQ maintained a high diagnostic performance for predicting the presence of TGCT, with an AUC of 0.862 (95% CI 0.724–1.00; *P* = 0.001). The optimal cut‐off value determined by the Youden index was an RQ of 5.27, corresponding to a sensitivity of 100% and a specificity of 67%.

In binary logistic regression, a positive miR371a result was a strong independent predictor of TGCT (OR 10.67, 95% CI 2.72–41.84; *P* = 0.001). Tumour size alone demonstrated a moderate predictive value (OR 2.06, 95% CI 1.16–3.63; *P* = 0.013; Nagelkerke *R* ^2^ = 0.193) but lost statistical significance when included alongside miR371a (*P* = 0.081). The combined model explained 43.5% of the variance (Nagelkerke *R* ^2^) and achieved a correct classification rate of 78.3%.

After the initial preoperative miR371a measurement, we also analysed the postoperative miR371a serum concentration in a total of 20 patients in our TGCT cohort. In paired analysis, postoperative miR371a levels were significantly lower than preoperative values (Wilcoxon *P* = 0.011), with a median decrease of 208 RQ units (95% CI –668 to −8.7).

## Discussion

In this single‐centre real‐world cohort, the CE‐certified miR371a assay achieved a sensitivity of 77.5% and a specificity of 80% for distinguishing TGCT from benign intratesticular lesions in patients with STM‐negative masses. These values are lower than those reported in large multicentre cohorts, where sensitivities frequently exceed 90%. This discrepancy is largely attributable to differences in tumour spectrum: our cohort consisted mainly of small, clinical Stage I, predominantly STM‐negative seminomas, a subgroup known for low miR371a expression. Previous studies have shown sensitivity increasing from ~59% in seminomas <1 cm to around 77% in lesions measuring 1–2 cm [13]. All false‐negative seminomas in our series were small (mean diameter 1.4 cm). Only one NSGCT was missed, a tumour with 85% teratoma content, consistent with the absence of miR371a expression in teratoma [14].

Specificity differences across studies similarly reflect variations in case mix. Earlier reports often included healthy volunteers or benign scrotal conditions [9, 15], yielding higher specificities than expected in routine clinical practice. In contrast, our control group consisted exclusively of sonographically suspicious intratesticular lesions, a diagnostically challenging comparator in which low‐level miRNA signals may occur despite benign histology. This design reflects real‐world uncertainty and provides a more realistic estimate of assay performance.

The diagnostic threshold has a major impact on test characteristics. Following the CE‐certified manufacturer recommendation, we defined RQ ≥10 as positive. Applying the lower cut‐off of RQ ≥5 used by Dieckmann et al. [9] would have increased sensitivity in our cohort to 95% overall and 97% in seminomas, as most false‐negative seminomas fell within the intermediate range of RQ 5–10. However, this came with a substantial loss of specificity (60%). Our ROC analysis identified an optimal threshold of 11.08, suggesting that a slightly higher cut‐off may yield more balanced performance in real‐world settings. In the meta‐analysis by Zhao et al. [16], eight of 11 studies reported miR371a cut‐off values, most derived *post hoc* by ROC analysis, which optimises test performance within a dataset but limits generalisability.

A structured indeterminate zone with mandatory re‐testing, as proposed by Lafin et al. [17], may improve reproducibility by identifying borderline RQ values within the intermediate range prone to stochastic amplification effects. Applying such an approach in our cohort might have corrected several borderline false‐negative seminomas and false‐positive benign lesions.

Routine clinical implementation is further constrained by logistical dependencies, including strict temperature control and processing times, which currently limit widespread adoption [18].

Comparison with the S1STeM‐371 study (ClinicalTrials.gov identifier: NCT04435756) highlights the impact of methodological factors. Although both cohorts showed a similar proportion of GCTs (66% vs 65.6%) and comparable distributions of small tumours, sensitivity at the CE‐certified cut‐off RQ ≥10 differed markedly [19].

The most plausible explanation is pre‐analytical handling: in S1STeM‐371, serum was processed on‐site within a tightly standardised workflow, whereas all samples in our real‐world setting required transport to the molecular pathology laboratory. Despite meeting the 2‐h processing window, transport likely introduced mechanical stress and temperature fluctuations. Together with operator variability, these factors may have increased analytical noise, particularly in the RQ 5–10 range where most of our false‐negative results occurred.

Even minimal pre‐analytical deviations can shift circulating miRNA measurements by one to two PCR cycles [12], potentially lowering true‐positive small‐volume tumours below the diagnostic threshold. Platform‐dependent variability at low RQ values is also possible. Importantly, our study used the LightCycler 480 II, whereas S1STeM‐371 performed all measurements on the QuantStudio 12 K Flex (Thermo Fisher Scientific, Waltham, MA USA), introducing an additional layer of technical variation despite both platforms being validated for the mir|detect workflow [20].

Using the liberal cut‐off RQ ≥5, our cohort achieved a sensitivity of 95% at a specificity of 60%, compared with 100% sensitivity and 45% specificity in S1STeM‐371. Owing to small sample sizes, performance estimates in both studies remain highly sensitive to borderline cases. Harmonised analytical procedures and multicentre validation may reduce such variability.

Future directions include more sensitive approaches for detecting very low copy numbers of circulating miR371a and improved stabilisation of blood samples, which may enhance sensitivity and enable wider implementation in smaller centres without access to specialised molecular pathology.

All STM‐positive tumours were miR371a‐positive (100%), and among STM‐negative carcinomas the assay detected 23 of 32 lesions (sensitivity 72%). All false positives occurred in STM‐negative benign lesions, resulting in a specificity of 80%. Overall, miR371a provided diagnostic added value in ~70% of STM‐negative cases.

Neither clinical nor sonographic features reliably distinguished benign from malignant small tumours, and tumour size alone offered limited discriminatory power. In regression analysis, size predicted malignancy in univariable testing but lost significance once miR371a was included, indicating superior discriminative ability of the molecular marker. The combined model explained 43.5% of the variance and correctly classified 78.3% of cases.

The miR371a assay particularly improved risk stratification in STM‐negative lesions, where conventional markers are uninformative. Our findings support more selective management of small, impalpable, STM‐negative tumours. In carefully selected patients, especially those ≤2 cm and miR371a‐negative, active surveillance may be reasonable. However, reduced sensitivity in very small seminomas and the inability to detect teratoma necessitate cautious application and structured follow‐up.

Taken together, these findings suggest that translation of miR371a testing into large‐scale routine practice requires rigorous methodological control to ensure reproducible performance. Experience from real‐world settings indicates that variability outside tightly standardised conditions may influence test characteristics.

At the same time, the assay may be particularly valuable in small, STM‐negative intratesticular masses, where conventional markers provide limited guidance and improved molecular stratification could meaningfully inform management decisions.

## Strength and Limitations

Our study has several strengths and limitations that must be considered before general recommendations.

A major strength of our study is its focus on a real‐world preoperative cohort of patients with clinically suspicious, STM‐negative intratesticular lesions, reflecting the population in which miR371a testing could most influence management. The use of a CE‐certified, standardised assay ensures regulatory‐grade reliability and enhances reproducibility.

However, this study is limited by its retrospective, single‐centre design, and modest sample size, which may restrict generalisability and introduce selection bias. Intermediate RQ results (i.e., 5–10) were pragmatically classified as negative due to logistical constraints, potentially underestimating sensitivity. Although the CE‐certified assay strengthens methodological robustness, its limited availability and technical demands currently restrict widespread clinical adoption, particularly in smaller centres and outpatient settings.

As tumour size is usually a subgroup analysis in larger study populations, further prospective studies of small STM‐negative testicular masses including miR371a are needed to provide sufficient data on the sensitivity and specificity of miR371a in these small tumours.

Although postoperative miR371a kinetics are of high clinical interest, our study was not designed or powered to evaluate clearance dynamics or recurrence monitoring. Sampling was available only at a very early postoperative timepoint, and follow‐up data were insufficient for meaningful interpretation. These aspects warrant dedicated prospective investigation.

## Conclusion

In this single‐centre pilot study, the CE‐certified miR371a assay provided meaningful diagnostic added value for distinguishing TGCTs from benign intratesticular lesions in a real‐world preoperative cohort. Standardisation of thresholds, pre‐analytical handling, and analytical procedures will be essential to ensure reproducibility and to facilitate broader clinical implementation. Multicentre prospective validation is needed to define its optimal role in clinical decision‐making and to support wider adoption. Future studies should apply a structured diagnostic framework incorporating pre‐defined hypotheses, repeated testing of intermediate results, harmonised pre‐analytical handling, and prospective cut‐off validation across multiple centres.

## Disclosure of Interests

Felix Seelemeyer declares no conflicts of interest. David Pfister declares no conflicts of interest. Ruben Gößmann declares no conflicts of interest. Olivia Steenbock declares no conflicts of interest. Roberto Pappesch declares no conflicts of interest. Sabine Merkelbach‐Bruse declares no conflicts of interest. Axel Heidenreich declares no conflicts of interest.

## Author Contributions

Felix Seelemeyer: study conception and design, data collection, data analysis, manuscript drafting. David Pfister: patient recruitment, data interpretation, critical revision of the manuscript. Ruben Gößmann: data interpretation, critical revision of the manuscript. Olivia Steenbock: data collection. Roberto Pappesch: molecular pathology analyses, data interpretation. Sabine Merkelbach‐Bruse: supervision of molecular analyses, methodological input. Axel Heidenreich: supervision, study design, critical revision of the manuscript.

## References

[1] Krege S , Albers P , Heidenreich A . The role of tumour markers in diagnosis and management of testicular germ cell tumours. Der Urologe Ausg A 2011; 50: 313–321 21327901 10.1007/s00120-010-2414-5

[2] Murray MJ , Huddart RA , Coleman N . The present and future of serum diagnostic tests for testicular germ cell tumours. Nat Rev Urol 2016; 13: 715–725 27754472 10.1038/nrurol.2016.170

[3] Fankhauser CD , Roth L , Kranzbühler B et al. The role of frozen section examination during inguinal exploration in men with inconclusive testicular tumors: a systematic review and meta‐analysis. Eur Urol Focus 2021; 7: 1400–1402 32684510 10.1016/j.euf.2020.06.019

[4] Gentile G , Rizzo M , Bianchi L et al. Testis sparing surgery of small testicular masses: retrospective analysis of a multicenter cohort. J Urol 2020; 203: 760–766 31580179 10.1097/JU.0000000000000579

[5] Wardak S , Pang KH , Castiglione F et al. Management of small testicular masses: outcomes from a single‐centre specialist multidisciplinary team. BJU Int 2023; 131: 73–81 35986901 10.1111/bju.15874

[6] Paffenholz P , Held L , Loosen SH , Pfister D , Heidenreich A . Testis sparing surgery for benign testicular masses: diagnostics and therapeutic approaches. J Urol 2018; 200: 353–360 29530784 10.1016/j.juro.2018.03.007

[7] Sharbidre KG , Lockhart ME . Imaging of scrotal masses. Abdominal Radiology 2020; 45: 2087–2108 31919649 10.1007/s00261-019-02395-4

[8] Das MK , Haugen ØP , Haugen TB . Diverse roles and targets of miRNA in the pathogenesis of testicular germ cell tumour. Cancer 2022; 14: 1190 10.3390/cancers14051190 PMC8909779 35267498

[9] Dieckmann KP , Radtke A , Geczi L et al. Serum levels of MicroRNA‐371a‐3p (M371 test) as a new biomarker of testicular germ cell tumors: results of a prospective multicentric study. J Clin Oncol 2019; 37: 1412–1423 30875280 10.1200/JCO.18.01480 PMC6544462

[10] Myklebust MP , Thor A , Rosenlund B et al. Serum miR371 in testicular germ cell cancer before and after orchiectomy, assessed by digital‐droplet PCR in a prospective study. Sci Rep 2021; 11: 15582 34341387 10.1038/s41598-021-94812-2 PMC8329070

[11] Belge G , Grobelny F , Radtke A et al. Serum levels of microRNA‐371a‐3p are not elevated in testicular tumours of non‐germ cell origin. J Cancer Res Clin Oncol 2021; 147: 435–443 33200255 10.1007/s00432-020-03429-x PMC7817581

[12] Ye F , Feldman DR , Valentino A et al. Analytical validation and performance characteristics of molecular serum biomarkers, miR‐371a‐3p and miR‐372‐3p, for male germ cell tumors, in a clinical laboratory setting. J Mol Diagn 2022; 24: 867–877 35934321 10.1016/j.jmoldx.2022.04.007 PMC9379668

[13] Nestler T , Schoch J , Belge G , Dieckmann KP . MicroRNA‐371a‐3p‐the novel serum biomarker in testicular germ cell tumors. Cancer 2023; 15: 3944 10.3390/cancers15153944 PMC10417034 37568759

[14] Dieckmann KP , Isbarn H , Grobelny F et al. Testicular neoplasms: primary tumour size is closely interrelated with histology, clinical staging, and tumour marker expression rates‐a comprehensive statistical analysis. Cancer 2022; 14: 5447 10.3390/cancers14215447 PMC9653836 36358866

[15] Liu Q , Lian Q , Lv H , Zhang X , Zhou F . The diagnostic accuracy of miR‐371a‐3p for testicular germ cell tumors: a systematic review and meta‐analysis. Mol Diagn Ther 2021; 25: 273–281 33886084 10.1007/s40291-021-00521-x

[16] Zhao XY , Gao YL , Li DF , Liu HC , Zhu RF , Zhu CT . Diagnostic performance of microRNAs in testicular germ cell tumors: a systematic review and meta‐analysis. Aging 2021; 13: 19657–19677 34343969 10.18632/aging.203376 PMC8386578

[17] Lafin J , Scarpini C , Amini A et al. Refining the serum miR‐371a‐3p test for viable germ cell tumor detection: identification and definition of an indeterminate range. Res Sq 2023; rs.3: rs‐2644890 10.1038/s41598-023-37271-1 PMC10310745 37386046

[18] Timmerman DM , Gillis AJM , Mego M , Looijenga LHJ . Comparative analyses of liquid‐biopsy MicroRNA371a‐3p isolation protocols for serum and plasma. Cancer 2021; 13: 4260 10.3390/cancers13174260 PMC8428229 34503070

[19] Nazzani S , Busico A , Bernasconi V et al. Clinical evaluation of the role of miRNA 371 in small testicular masses. Results of the “S1STeM 371” trial. Eur J Cancer 2025; 223: 115494 40382859 10.1016/j.ejca.2025.115494

[20] GmbH md . Gebrauchsanweisung M371‐Test Version 14. Bremerhaven, Germany: mir, 2025

